# Albinism and Blood Cell Profile: The Peculiar Case of Asinara Donkeys

**DOI:** 10.3390/ani14182641

**Published:** 2024-09-11

**Authors:** Maria Grazia Cappai, Alice Senes, Giovannantonio Pilo

**Affiliations:** 1 Nutrition Desk of the Veterinary Teaching Hospital, University of Sassari, 07100 Sassari, Italy; a.senes1@studenti.uniss.it; 2 Istituto Zooprofilattico Sperimentale della Sardegna “G. Pegreffi”, 07100 Sassari, Italy; gantonio.pilo@izs-sardegna.it

**Keywords:** albino, anemia, anisocytosis, antioxidant, equine, erythrocyte, hemoglobin, melanocyte, metabolism

## Abstract

**Simple Summary:**

Asinara donkeys are unique worldwide for expressing a particular form of albinism spread to all individuals of the breed. This peculiar condition of oculo-cutaneous albinism of type 1 (OCA1) is a rare condition from a recessive autosomic mutation, fixed through generations, thanks to the isolation of donkeys on Asinara Island (from which the breed name originates) favoring inbreeding. Asinara island is a small island of Sardinia, one of the biggest islands in the Mediterranean sea. Asinara donkeys have shown good adaptation to the Mediterranean climate through various metabolic strategies, one of which relies on endogenous retinol mobilization during the positive photoperiod for protective effect and adequate circulating vitamin E. Indeed, their exposure to sun radiation and high temperature on one hand, and the lack of melanin in the coat, skin, iris and mucocutaneous junctions on the other, require them to overcome such an extended lack of photoprotective pigment (melanin) in alternative ways to prevent UV radiation damage. In this investigation, our interest was oriented toward screening the complete blood cell count to understand whether metabolic adaptation to the environment involved blood cell lines. Such an idea comes from the fact that in humans, a particular condition of albinism is reported to share the same gene with other mutations involving hemoglobin synthesis. In our case, significant differences were observed in relation to red blood cells (RBCs), in terms of their average volume (MCV), width distribution (RDW-CV), and width deviation (RDW-SD), in comparison to pigmented (gray) Sardo donkeys.

**Abstract:**

The complete blood cell count (CBC) was screened in a group of 15 donkeys, of which 8 were of Asinara breed (oculocutaneous albinism type 1, OCA1) and 7 of Sardo breed (gray coat). All donkeys were kept under same management and dietary conditions and underwent periodic health monitoring in the month of June 2024, at the peak of the positive photoperiod, at Mediterranean latitudes. One aliquot of whole blood, drawn from each individual into K_2_-EDTA containing tubes, was analyzed for the complete blood cell count through an automatic analyzer, within two hours of sampling. Data were analyzed and compared by one-way ANOVA, where the breed was an independent variable. All animals appeared clinically healthy, though mild eosinophilia was observed in Sardo donkeys. The red blood cell line showed peculiar traits for Asinara donkeys, which displayed significantly higher circulating red blood cell numbers than gray coat Sardo donkeys (RBC, 5.19 vs. 3.80 10^12^/mL ± 0.98 pooled-St. Dev, respectively; *p* = 0.017). RBCs also exhibited a smaller diameter and higher degree of anisocytosis in Asinara donkeys, along with lower hematocrit value, albeit within physiological ranges. Taken all together, such hematological profile depicts a peculiar trait of the red blood cell line in albino donkeys during the positive photoperiod.

## 1. Introduction

**Figure 1:**
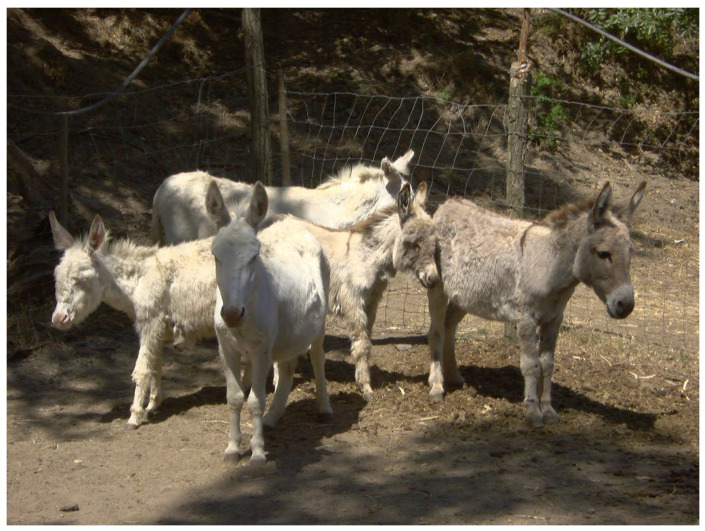
Group of Asinara (albino) and Sardo donkeys (gray) together. Jennies with filly and colt.

The albinism condition is characterized by different forms expressed in the phenotype, following the biochemical impairment in different phases of the melanogenic process, stopped at the earliest steps due to enzymatic inactivity [1,2,3,4]. Melanin is a natural pigment produced by melanocytes, which are highly specialized cells derived from the neural crest, distributed in the basal layer of the epidermis [5,6]. Though not only melanocytes produce melanin in the animal body, it is also important to point out that the albinism condition in Asinara donkeys is due to a genetic mutation of the TYR Gene encoding for the tyrosinase enzyme (*Tyr*): partial or total enzymatic inefficiency of tyrosinase can in fact lead to different albino forms, whatever the embryogenic origin of the cells with the ability to synthesize melanin. Melanin is a pigment produced across the animal kingdom, and is responsible for the coloration of the skin, as well as that of hairs, feathers, scales, iris, and mucosa of the mucocutaneous junctions [7]. In humans, different subtypes of albinism are described in the literature and different genes have been identified as being involved. Moreover, according to the albinism subtype, syndromic and non-syndromic forms are also acknowledged in albino individuals [8]. Melanin production is genetically encoded and elicited according to external stimuli. In fact, among its biological roles, photoprotection against UV radiation can be considered as chiefly important. One main action of melanin molecule is to behave as an endogenous antioxidant, taking part in limiting the damage of tissues exposed to direct sunlight [9,10]. Despite albinism being reported in reptiles, amphibians, birds, fish, and mammals, such occurrences are usually described as being limited to individual cases [11,12,13,14,15,16,17,18,19]. The peculiar form of oculocutaneous albinism of type 1 (OCA1) in Asinara donkeys is spread to all individuals of the breed [1]. The genetic mutation encoding for the tyrosinase enzyme (involved in the first steps of the melanogenic process) was fixed through generations, thanks to geographical isolation, favoring an inbreeding phenomenon [20]. The albinism of Asinara white donkeys has been recently identified to be due to a missense mutation in a highly conserved amino acid position (G/G or D/D genotype), diverse from the pigmented phenotype (gray) of the Sardo donkey (C/C or C/G genotype) [7]. Asinara donkeys living on the Asinara island (from which the breed’s name originates), a small isle of Sardinia in the Mediterranean Sea, likely branched out of the gray Sardo donkey back in time, in which specimens can carry the gene for this autosomal recessive mutation [21] (Figure 1). The exposure to sunlight during the positive photoperiod at Mediterranean latitudes could be extremely challenging. Nevertheless, previous investigations pointed out the marked coping ability of Asinara donkeys to prolonged and intense sunlight exposure. If adaptation is accounted for, the metabolic response developed by Asinara donkeys is noteworthy and relies on the high circulating levels of retinol (in retinyl-esters form) along with tocopherol, as fat soluble vitamins with strong antioxidant activities, supporting endogenous antioxidant systems [22,23]. Such metabolic adaptation proves these animals as examples of successful fitness to the environment, and as an outstanding resilient feral breed (Figure 1). However, both exogenous (dietary fat soluble vitamins) and endogenous antioxidant systems may be tested by the augmented request of photoprotection, leading to expected seasonal and systemic increases in oxidative stress. In addition, circulating tocopherol in the bloodstream of Asinara donkeys may reflect a readily available source of antioxidants on one hand. But, along with assuring antioxidant support drained by the integument following the lack of melanin, it could be hypothesized that requirements for antioxidant support by other tissues may also increase, likely involving erythrocytes, among other cells. It is known that tocopherol is an integral molecule of the cell membrane, and of course, of that of red blood cells [24,25,26,27]. Red blood cells (RBCs) are characterized for being among the smallest cells of the animal body and typically lacking a nucleus in mammals. Their peculiar plasticity to pass through capillaries and allow gas exchange requires a high cell surface area in relation to the cell volume and a peculiar metabolism (pentose phosphate pathway, leading to NADPH). Thus, the stability of the cell membrane of erythrocytes is of extreme importance, in view of the mechanical solicitation and oxidative stress. It is also known that human forms of albinism share gene 11 with a mutation encoding for abnormal hemoglobin synthesis (HGBs), causing the so-called sickle cell anemia [28]. Such a co-morbidity ruled by autosomal and recessive mutations on human gene 11 is described in the sub-Saharan population, where high consanguinity was postulated, and where intense sunlight exposure is a co-factor. Additionally, other co-occurrences of correlated diseases were also associated with the albinism phenotype [29].

In view of this scenario, the question about the potential correlation between the albinism condition and metabolic adaptation in Asinara donkeys living at Mediterranean latitudes needs to be elucidated further. The existing literature is scanty about such aspects, to the best of our knowledge. Other cases of albinism in donkeys are excluded [30], for white coat is present in this species. The study of the hemochrome and cytometry profile in apparently healthy Asinara donkeys seemed to be highly stimulating, and reasonably supported by the actual knowledge on albinism. Thus, it was hypothesized that the evaluation of the complete blood cell count (CBC) in Asinara donkeys could be informative as to whether phenotypic differences could be pointed out. As a first step, we aimed to compare the hematological profile between specimens of Asinara and Sardo breeds, under identical keeping condition. To emphasize potential differences, all animals were screened during the positive photoperiod in the month of June 2024, when the sun radiation exposure is highest. The specific objective was to assess if any differences in the CBC and related indices could be observed, potentially associated with the oculocutaneous albino (OCA) phenotype of Asinara donkeys.

## 2. Materials and Methods

### 2.1. Animal Care

All animals involved in the present screening were handled in compliance with recommendations of European Union directive 86/609/EEC and Italian law 116/92 concerning animal care. Donkeys under this screening activity are not experimental animals, and were not enrolled in an experimental protocol, but underwent periodic health monitoring for serological analyses and surveillance of diffusive infections, for the screening of Equine Infectious Anemia (EIA) via Coggins test.

### 2.2. Animal Keeping

A drove of donkeys (1 colt; 1 filly; 2 jacks; 12 jennies) was screened in this investigation. All animals lived together in the same private farm (Latitude 40°44′15.48″ N, Longitude 8°27′51.4″ E, Altitude 92 m a.s.l.) and shared pasture, facilities, and feeding practices. All animals were assessed by the official veterinary surgeon for clinical conditions before blood sampling for epidemiological surveillance of EIA. For the specific purposes of this investigation, all animals underwent nutritional assessment. Individual clinical and nutritional assessment [31] and sampling involved 15 donkeys, of which 8 were of Asinara and 7 of Sardo breed: each animal was individually recorded in the Official Record of local minor breeds as white donkey of Asinara (Ministerial Decree 27 July 1990) or Sardo breed, respectively, and electronically identified with unique number of transponder (EU Regulation 2015/262).

All animals had free access to spontaneous pasture during the day. During the nighttime, they were sheltered in the barn. Feeding practices consisted of the administration of forage (hay) and of compound mixed feed for horses at maintenance, consistently with an estimation of the body condition of the donkeys. All the donkeys are kept as companion animals.

### 2.3. Sampling and Laboratory Procedures

Each donkey from both breeds was sampled for whole blood to carry out the serological test for EIA via Coggins test. For this purpose, all animals were gathered and sampled individually. Whole blood was drawn through the puncture of the jugular vein into 9 mL vacuum tubes. One aliquot was collected in 9 mL vacuum tubes (Vacutainer Systems Europe; Becton Dickinson, Meylan Cedex, France) containing ethylenediaminetetraacetic acid dipotassium salt dehydrate (K_2_-EDTA) as anticoagulant. Individual blood containing tubes were classified with labels (EIC, breed, date). Tubes were held in the upright position through polystyrene cases and taken in a refrigerated bag (cooled by ice blocks in box for blood sample storage and transport) to the laboratory. Processing of whole blood samples was initiated within 2 h of collection. The following red blood cell (RBC) indices were considered: (1) MCV: defines the size of the red blood cells and is expressed as femtoliters (10–15; fl); (2) MCH: quantifies the amount of hemoglobin per red blood cell and is expressed in picograms (pg); (3) MCHC: indicates the amount of hemoglobin per volume unit, and in contrast to MCH, MCHC correlates the hemoglobin content with the volume of the cell and is expressed as g/L of RBCs; (4) RDW-SD: represents the coefficient of variation of the RBC volume distribution (size) and is expressed in femtoliters. It is a good indicator of the degree of anisocytosis.

Individual samples were placed on an automatic tube agitator before being processed on an automatic analyzer for complete blood cell count (Alcyon BC2800Vet Mindray). Each sample was also tested on a smear test to exclude the occurrence of accidental platelet clumping or undesired RBC aggregation (rouleaux), preventing the sample from being considered as diagnostic. Briefly, one drop of whole blood was collected from each tube with the help of single use sterile polypropylene loop (10 μL) and placed on one edge of a clean antistatic glass slide. The drop of blood was then smeared over the surface of the slide until monolayer cell, gently obtained with a spreader (thin glass cover), stained with a May-Grünwald Giemsa kit and observed under light microscope.

### 2.4. Data Analysis and Statistics

Hematological data were analyzed for variance between the two breeds (Asinara vs. Sardo) via one-way ANOVA, by running a datasheet on Minitab_19 © (State College, PA, USA).

The statistical significance was set for *p*-value < 0.05. A *p*-value < 0.10 represented a trend.

## 3. Results

All animals involved in the trial appeared healthy and tested negative to Coggins test. No sign of nutritional deficiency could be pointed out in either breed at nutritional assessment, and adequate body condition was also found [BCS, 1–9-point scale: 5 to 6, normal weight].

The CBC confirmed the healthy condition of all animals, except for mild eosinophilia in 6 out of 15 donkeys, of which 83.3% belonged to the Sardo breed (*p* = 0.022), with absolute [EOS (10^9^/L): 1.16 ± 0.29, normal range 0.01–1.00] and relative values [%EOS: 0.10 ± 0.02, normal range 0.01–0.08] of white blood cell count. The hemogram showed significant differences between the two breeds as the cell count and derived indices were considered. Despite the differences being significant, values were within the range considered normal for this species. Circulating RBC turned out to be significantly higher in number (*p* = 0.017) and smaller in volume (*p* = 0.006) in Asinara donkeys than in Sardo breed donkeys. In addition, a significantly higher (*p* = 0.048) level of anisocytosis was reported in Asinara donkeys (RDW-CV:0.20 ± 0.01) than in Sardo donkeys (RDW-CV: 0.18 ± 0.00). Microscopic visualization of blood smears allowed us to consider all samples as diagnostic. Some isolated cases of eccentrocyte were found, as a sign of oxidative damage. The mean cell hemoglobin (MCH) was significantly lower (*p* = 0.005) in Asinara donkeys, with a comparable concentration (MCHC) in both breeds and higher hematocrit (HCT) in Asinara than in Sardo donkeys (which appeared slightly below the lower limit of the physiological range) in a non-significant way. Higher levels of RBCs contribute to the higher hematocrit value in Asinara donkeys, though also in the physiological range for the species, being close to a trend (*p* = 0.106). The index related to red blood cell distribution width was significantly higher in Asinara donkeys in which higher level of anisocytosis was found. 

The data are reported in Table 1.

The platelet number per volume unit turned out to be constantly higher than the upper value considered to be physiological for the species, in both Sardo and Asinara donkeys. Platelet indices did not display significant differences between specimens of the two breeds. In fact, the mean platelet volume (MPV, *p* = 0.796), plateletcrit (PCT, as expression of mean platelet volume × platelet concentration, *p* = 0.370), and platelet diameter width (PDW, *p* = 0.820) did not substantially differ among all animals.

**Figure 2:**
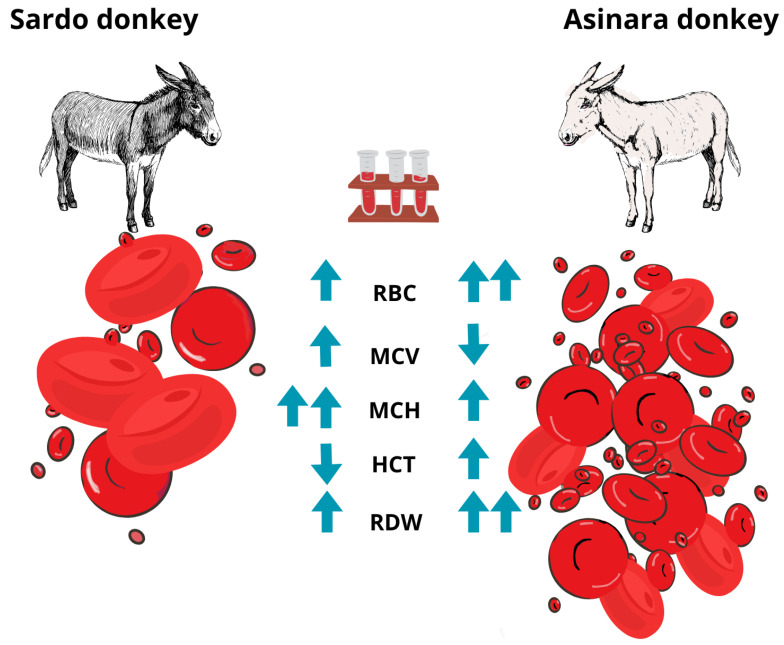
Sardo and Asinara breed specimens showing the different phenotype and CBC.

The pattern of the significant difference is illustrated in Figure 2.

## 4. Discussion

The complete hemogram of Asinara donkeys was determined here for the first time. Although the number of specimens may appear low to interpret results exhaustively in a definitive way, it is important to underline that the screening was conducted on animals that display the albino phenotype spread to all individuals of the breed. The possibility to screen the potential implication of OCA1 on groups of animals is a rare condition, because the literature reports data on albino individuals, regardless of whether it is an animal or human case description. In addition, the chance to compare the CBC of Asinara donkeys to that determined in Sardo donkeys, kept under identical conditions in the same farm and at the same time, supports the data obtained in this screening. The interpretation of the analyses on blood cell lines should also account for the fact that the positive photoperiod at Mediterranean latitudes is characterized by a high temperature (not rarely over 40 °C in the hottest hours of the day) and the length of daylight hours reaching up to 16 h, with extreme climate conditions. 

The lack of melanin can therefore be a serious health problem, above all when endogenous antioxidant systems are tested in the hot season. In addition, the co-occurrence of other diseases was recently reviewed from clinical case descriptions of human subjects with different albino forms [29]. The implications of albinism in wild and feral animals is worthy of being studied in detail, though animals screened here were client-owned. 

Indeed, moving from the description reporting co-morbidity in humans, the hypothesis of the involvement of the RBC line in Asinara donkeys was correct in this case. In fact, the outcome of our analyses displays a pattern. The peculiarity of the hemogram is supported by the limited variability among individuals. In fact, with the sole exception of a mild increase in EOS in Sardo donkeys, the values of CBC were within the range considered normal for the species [32,33] for all the animals. However, when the breed was used as an independent variable, significant differences were observed in relation to a higher circulating RBC number, smaller MCV, higher MCH, higher HCT, and higher RDW-CV and RDW-SD in Asinara donkeys, in comparison with those obtained from Sardo donkeys. The combination of indices and levels of RBC offers several considerations. Erythrocytosis along with a high level of anisocytosis and small diameter of RBC seem to point to an anemic condition. The platelet profile gathered our interest and appears worthy of being explored further, even though the associated indices did not highlight differences between the breeds at this stage and were not of clinical importance. The MCH appears lower in Asinara (along with having a higher number of circulating RBC, being smaller in volume), but the MCHC is comparable between the two breeds. Although further analyses should be carried out, the peculiar morphology of RBC in Asinara donkeys encourages further investigation of the hemogram, with a special focus on HGB, along with the coagulation profile.

## 5. Conclusions

Albino donkeys of Asinara breed show a peculiar profile of CBC at the comparison with Sardo breed donkeys kept under same farming conditions. The hemogram is characterized by erythrocytosis and anisocytosis likely resembling a sub-clinical anemic condition. The results obtained in this screening are encouraging and warrant further investigation of the role of HGB in detail.

## Figures and Tables

**Table 1:** Hemogram of individuals (Sardo vs. Asinara) and significance.

Item ^1^	Reference Values ^2^	Breed		Statistics	
		Sardo	Asinara	Pooled-St Dev	*p*-Value
NEUT (10^9^/L)	2.40–6.30	5.49	4.59	1.83	0.361
LYMPH (10^9^/L)	2.20–9.60	4.55	8.02	2.26	0.011
MONO (10^9^/L)	0.00–0.75	0.51	0.56	0.17	0.568
EOS (10^9^/L)	0.10–0.90	1.16	0.74	0.33	0.022
BASO (10^9^/L)	0.00–0.07	0.03	0.03	0.01	0.846
WBC (10^9^/L)	6.20–15.0	11.7	13.9	2.73	0.144
RBC (10^12^/L)	4.40–7.10	3.80	5.19	0.98	0.017
HGB (g/L)	89.0–147	86.7	100.0	16.6	0.124
HCT (%)	24.3–39.6	23.3	27.3	0.47	0.106
MCV (fl)	53.0–67.0	61.8	53.8	4.74	0.006
MCH (pg)	17.6–23.1	22.9	19.7	1.82	0.005
MCHC (g/L)	310–370	371	366	7.46	0.250
PLT (10^9^/L)	95.0–384	448	414	73.2	0.391
RDW-CV	0.19–0.25	0.18	0.20	0.01	0.048
RDW-SD (fl)	35.5–45.8	40.3	42.2	2.11	0.052

^1^ Item: WBC = white blood cells; NEUT = neutrophils; LYMPH = lymphocytes; MONO = monocytes; EOS = eosinophils; BASO = basophils; NEUT= neutrophils; RBC = red blood cells; HGB = hemoglobin; HCT = hematocrit; MCV = mean corpuscular volume; MCH = mean cell hemoglobin; MCHC = mean corpuscular hemoglobin concentration; PLT = platelet; RDW-CV = red blood cell distribution width. ^2^ Reference values: adapted from [32,33].

## Data Availability

The raw data are available as a Supplementary File.

## Supplementary Materials

The following supporting information can be downloaded at: https://www.mdpi.com/article/10.3390/ani14182641/s1, Raw data and owner consents are available as supplementary material.
